# From Policy to Practice: A Qualitative Study on Reforms and Frontline Retention in Healthcare

**DOI:** 10.1177/00469580251365821

**Published:** 2025-08-16

**Authors:** Anik Dubé, Stéphanie Collin, Jennifer Hakim, Claire Johnson, Marie-Eve Laforest, Michel H. Landry, Martin Lauzier

**Affiliations:** 1 Université de Moncton, NB, Canada; 2 Université de Sherbrooke, Moncton, QC, Canada; 3 Université du Québec en Outaouais, Gatineau, QC, Canada

**Keywords:** health care reform, physicians, delivery of health care, nurses, government, social responsibility

## Abstract

In Canada, healthcare reforms typically aim to improve the quality of care and access while making healthcare systems more efficient. These reforms have led to a 2-level healthcare system consisting of provincial and regional health authorities (RHAs). RHAs are responsible for providing and administering health services within specific territories. One of the 2 language-based RHAs in New Brunswick (NB) operates in French-speaking rural minority communities. This study explored key factors affecting the retention of nurses and physicians within a RHA operating in a language minority context. This descriptive qualitative study explored how macro-level decisions are experienced on the frontlines. Data were collected through semi-structured interviews with 21 physicians and 37 registered nurses, as well as 2 focus groups involving 20 key informants in managerial roles. Thematic analysis was used to identify key themes. Three main factors emerged: organizational accountability and frustration, local autonomy and contextual responsiveness, and a culture of openness and perceived loss of control. These factors are associated with policy changes that affect operational settings and resource distribution within the RHA and influence the retention of nurses and physicians. Stakeholders in health system reforms, including governments and RHAs, must recognize that policy adjustments can have direct implications on everyday care. Participants expressed a growing disconnect from decision-making hierarchies and a perceived loss of control. Both are seen as barriers to delivering quality care. Ensuring adequate support and resources for implementing system-level changes is key to fostering professional engagement and enhancing job satisfaction.

## Highlights

- Findings show that administrative overload undermines care, and bureaucratic complexity increase workloads and reduce professional satisfaction.
- Results indicate that top-down reforms reduce local autonomy and hinder responsiveness to regional healthcare needs.
- Effective reform requires co-creation, collaboration, and adaptation to community-specific contexts.

## Introduction

Governments worldwide have made deliberate attempts to address health system dysfunction and respond to policy challenges such as escalating costs,^1-3^ poor population health outcomes,^4-6^ and health service inequalities.^7,8^ In Canada, structural health system reforms are intended to address persistent challenges identified by policymakers. These include regional disparities in healthcare services, inefficient patient flow, shortcomings in system planning, and overall, underperformance.^9,10^ However, such reforms have often led to the development of a 2-level healthcare system, raising concerns about the fragmentation of care. In the NB healthcare system, the central provincial authority typically plays a steering role by setting overarching objectives and allocating funding. While, RHAs are responsible for the delivery of healthcare services and programs to populations within specific territories.^11,12^

Existing literature indicates that restructuring efforts do not consistently achieve the desired outcomes.^13-16^ These initiatives have often failed to integrate community perspectives, often by centralizing power and reallocating resources away from local governance structures.^4,17,18^ Moreover, they have had minimal success in promoting service integration,^19,20^ and have generally led to stakeholder dissatisfaction.^15^ Some structural changes are disrupting the practices of healthcare professionals and prompting them to re-examine the value they place on their work.^15,21^ In NB, Canada’s only officially bilingual province, the language of healthcare delivery and the governance of institutions by the French-speaking minority play an important role in shaping the direction of healthcare reforms.^22^ Like other provinces and territories, the Francophone RHA strives to retain professional resources that are in short supply to ensure equitable access to healthcare services for the population. Although the issue of scarce resources has been exacerbated by the COVID-19 pandemic, the retention of healthcare professionals has been a subject of study for several years.^11,23,24^ Identified strategies to help policy makers retain healthcare professionals include removing barriers to their professional development,^24,25^ implementing initiatives that foster job satisfaction and work-life balance,^24^ providing mentoring programs for nurses and physicians,^26^ and securing political and executive commitment to these efforts.^27^ The consequences of policy change, in particular structural reforms, on the experience of healthcare professionals, and on their retention, have yet to be elucidated.

The primary objective of our study was to identify the factors influencing the retention of nurses and physicians in a RHA operating in a minority language setting. The findings from our study indicate that healthcare system reforms and structural reorganization can have an influence on frontline operations within the healthcare system.

## Methods

### Conceptual Framework

The *Magnet Hospitals* and *Making it work* frameworks provided a reference point, guiding the development of the questions used in the interviews and observations. These frameworks demonstrate how various internal and external factors within a RHA influence the retention of physicians and nurses. The *Magnet Hospitals* model, first introduced in a 1983 publication, revealed that organizations most successful in retaining nurses were action-oriented, driven by strong shared values, and had minimal hierarchical levels.^28-32^ While, the *Making it Work* framework suggests that providing team cohesion support influences the retention of health professionals in rural and remote areas.^33^

### Study Design

This study used a descriptive qualitative design to explore the structural factors within RHA and their impact on the retention of nurses and physicians. The study was conducted in collaboration with 1 of the 2 RHAs, the French language RHA, which is responsible for healthcare delivery across 4 specific health regions in NB. Table 1 provides details on the Canadian healthcare system, the province of NB and the RHAs.

**Table 1.** Characteristics and Description of the Canadian Healthcare System, New-Brunswick (NB) and Both of Its Regional Health Authorities (RHA).

Characteristics	Description	
Healthcare system funding in Canada	Predominantly publicly financed, with approximately 70% of health expenditures financed through general tax revenues. Structurally, it aligns more closely with the National Health Insurance (NHI) model observed in Australia.^10,34,35^	
Healthcare administration and structure	Each province and territory are responsible for administering universal hospital and primary care services. *Medicare* is known for the healthcare insurance plan for the publicly funded healthcare system.^35^	
Canada Health Act	The federal government’s five funding criteria: public administration, comprehensiveness, universality, portability, accessibility.^35^	
Healthcare delivery	Primary care in Canada is largely delivered through general practitioners (GPs) and nurse practitioners (NPs), who function as the initial point of contact for patients. Access to specialist care typically requires a referral from a GP or NP, a model that is consistently implemented across all Canadian provinces.^36^	
Population of NB	842 725^35^	
Unique features of the province of NB	NB is Canada’s only official bilingual province. Under the *Official Languages Act*, members of the public have the right to access healthcare services and programs provided in the official language of their choice, either French or English.^37^	
NB health system and structures since the 2008 reform	Two RHAs emerged, replacing the previous eight RHAs. One of the main goals was to ensure uniformity in service delivery across the bilingual province.^38^	
RHAs responsibilities	Provide health services to members of the public in the official language of their choice through the regional health authority’s health establishments, facilities, and programs.^37^	
Corporate names of the RHAs	Regional Health Authority A VHN	Regional Health Authority B HHN
Working language	French	English
Characteristics of the population served^29^	Primarily francophone	Primarily anglophone
	Significant number of rural communities in northern NB	Primarily urban areas and in southern NB
	Large proportion of elderly people	Younger population
	Health status below the Canadian average	Health status below the Canadian average
	Low socio-economic level	
Number of employees	7949 employees (VHN)^39^	14 000 employees (HHN)^40^

*Note*. HHN = Horizon Health Network; NB = New-Brunswick; RHA = Regional Health Authority; VHN = Vitalite Health Network.

The research team consisted of experts from diverse disciplines, including nursing, medicine, psychology, and healthcare management, bringing a wide range of perspectives to the study. This collaborative insight provided a comprehensive understanding of the barriers and facilitators affecting retention, considering the various organizational and practical healthcare realities related to context, roles, and professional experience. This study is part of a larger research project aimed at helping one of NB’s RHAs identify and implement strategies to promote the retention of physicians and registered nurses, taking into account the unique environmental factors.^41^ Ethics approval was obtained from the RHA (60584) and the university (2122-105), and written informed consent was obtained from participants for the semi-structured interviews and group discussions before data collection began.^27^

### Study Site and Participants

New-Brunswick (NB), a Canadian province, has 2 RHAs (see Table 1). Their collective mandate is to manage and deliver quality healthcare services across 7 provincial health regions in both the official languages (English and French). This province has a significant proportion of its population living in rural areas primarily served by the French RHA. While most healthcare services are concentrated in urban areas (51%), a substantial portion of the population (49%) resides in rural regions where access to healthcare is often more challenging and varies between regions and communities.^42^

### Sampling and Recruitment

A purposive sampling technique was used to gain a deeper understanding of the factors influencing retention among nurses and physicians in different healthcare settings and regions. The study included nurses and physicians who were currently working or had left the RHA within the past 5 years.

For the semi-structured interviews, 58 interviews were conducted among 21 physicians and 37 registered nurses across the 4 health zones of the study’s RHA. From this list, 4 people were unable to participate owing to time constraints. The sample consisted of 12 men and 46 women. Forty-one participants were currently employed and 17 left the RHA. Gender representation was consistent with NB’s present provincial healthcare workforce landscape. Doctors and nurses known to the research team were initially approached by email to participate in the study in the summer of 2022. The snowball recruitment technique^43^ was used to identify additional potential participants during interviews and focus groups. The 2 focus group samples included 20 participants, each from the northeastern part of the province, who were healthcare leaders with various roles from all 4 zones of the study’s RHA, such as managers, directors, and program planners.

### Data Collection

The researchers optimized the data collection methods by employing a triangulated approach, incorporating document retrieval, focus groups, and semi-structured interviews. Field notes were maintained throughout the study. The aim of these various methods was to enable the research team to describe and understand the structural factors influencing retention as experienced by participants, while identifying possible links between them. Additionally, 95 regulatory documents, including provincial announcements, internal regulations, policies and procedures, annual reports, action plans, and articles related to the retention of nurses and physicians in NB, were retrieved and examined to collect and analyze data. In June and September 2022, focus groups consisting of two 60-min discussions led by 2 or 3 research team members were conducted in 2 health regions. Participants were invited to share their views on the main challenges they faced in retaining physicians and nurses as well as the efforts or measures implemented within the RHA to address these challenges. Finally, semi-structured interviews were conducted either face-to-face or online, using a distance media platform, from July 2022 to January 2023. All interviews lasted between 60 and 90 min and were conducted by 4 research team members in one of the participants’ official languages of choice. With participants’ consent, the interviews were recorded for transcription.

The interviews were guided by a 5-section grid with 11 open-ended questions. The guide was also pilot tested with 2 health professionals prior to commencing research. The initial section gathered socio-demographic data and provided an overview of the participants’ career paths. Participants were then invited to share their perspectives on various topics, including factors influencing their job satisfaction, their perceived influence over decisions affecting their work, workload and job demand, leadership dynamics within their team and the RHA, their level of autonomy to innovate in the care delivery, and the importance of language in their choice of employment.

### Data Analysis

A thematic approach was used for qualitative analysis.^44^ Data collection and analysis were simultaneously completed with respect to saturation to allow for a greater perspective on retention. Although descriptive, this approach has proven useful in a healthcare context, highlighting the various factors that contribute to changes in practice and organization from a healthcare professional’s perspective by establishing the different links between the different factors related to the roles of nurses and physicians. Nvivo11 supported the coding process, which was carried out iteratively and collaboratively. Initial first-level coding was conducted as a team, leading to the development of a shared codebook that guided subsequent analysis. Second- and third-level coding was done by 2 primary coders, who independently reviewed and analyzed the transcripts before comparing interpretations to ensure consistency. To enhance credibility, 2 participants completed member checking. Also, our study adhered to the Standards for Reporting Qualitative Research (SRQR) relevant to the EQUATOR Network reporting guidelines.^45^ The research team met regularly biweekly for 3-h sessions to advance the data analysis and validation processes. Final themes and subthemes related to retention were discussed and confirmed with the full research team. Decisions were documented through notes and memos to enhance rigor and trustworthiness.

## Results

Our analysis identified 3 major themes related to the factors influencing physician and nurse retention: (a) organizational accountability and frustration, (b) local autonomy and contextual responsiveness, and (c) a culture of openness and perceived loss of control. These themes are elaborated upon in the following paragraphs.

### Organizational Accountability and Frustration

Participants reported that the consolidation of RHAs and the resulting structural changes have negatively impacted accountability within the organization. Both nurses and physicians described difficulties navigating the hierarchical structures, which hinder collaboration and strain professional relationships. Some expressed skepticism regarding the legitimacy of certain higher-level decisions. As one nurse participant mentioned, “*(. . .) sector consolidation has led to cuts in local management, creating significant obstacles, and the lack of immediate specialist support from staff located a few hours away worsens the situation*” *(IIP508)*

A nurse illustrated the impact of administrative restructuring through a specific policy concerning reporting sick leave: “We received a notice that this person [in region x] was responsible for managing work rotations while the person in [another] region dealt with other issues [such as sick leave].” (IIP105) According to the participant-nurse, this regional policy seriously impeded the department’s operational and institutional efficiency, leading to increased workloads for remaining staff. The comment highlights the administrative overloads faced by frontline professionals, particularly concerning personal accountability and fragmented communication channels.

One day, when I called [the person in region x] to say I was sick, they said, “I'm not the *one* who handles sick leave; you have to contact the person in your region [region y].” So, I called the person from my area, and they said, “I'm not the *one* who handles sick leave; you’ll want to call the person in the other region x.” I replied, “I've already called you both.” It's problematic when they're not sure of their role; it creates difficulties for us. My colleagues never received a replacement that day. . . (IIP105)

A physician-participant shared his views and frustration regarding the administrative burden and the impact of escalating local problems to higher levels within the RHA:

If I'm being honest, I'd say that since regionalization, finding your way around the organizational system has become a lot more difficult. The structure seems much heavier, and although we are gradually seeing some improvements, it's still very difficult. The decision-making process often seems far and disconnected from the people directly involved, making it difficult to access information. It's also difficult to get information up to the upper management level. Everything is managed by the specialty sector rather than locally or by the health region, which represents a major challenge. However, I'm not saying that these are all drawbacks; there are advantages to this structure. It's a question of improving it. I believe that work is underway at regional level to solve these problems, but it remains a challenge and a source of frustration for many people, because many people come to see me about this. (MDP279)

The interviews revealed a strong sense of nostalgia among physicians for the period before the 2008 health system reform when there were 8 RHAs. The impact of the reform on professional practice varied between the physician and nurse participants. Physicians reported facing significant obstacles in implementing innovations or improving patient care due to bureaucratic constraints and administrative overload. Conversely, nurses described a loss of decision-making authority within their units because of restructuring. Some nurses also highlighted a notable lack of support and disconnection from professional associations and unions, contributing to increased burnout and compromised workplace safety. The bureaucracy and perceived lack of accountability within the healthcare system contribute to a sense among healthcare professionals that delivering adequate patient care is unattainable, which can impact their loyalty to the RHA. Our study reveals that mid-level managers share similar frustrations, reporting that bureaucratic challenges hinder their day-to-day decision-making. This is what emerged from a focus group:

Today, we sent in our requests over the Internet, then they gave us a nice list, then we fell in last place, and then we must fight to get it. I used to go and see such-and-such, I was competing against such-and-such who needed something urgently, so we'd probably have talked. Now I must fight if I want to get a machine, I must establish a strategy, or I must pull some strings and go through all kinds of patents to get it because the machine is too big, and we don't even know who runs the machine. You know, it's like, ah, this isn't my part, this is the part for, ah, this isn't me either, this is. (FG600)

### Local Autonomy and Contextual Responsiveness

Most participants advocated for a re-evaluation of this long-standing reform and proposed a hybrid regionalization model. This model retains certain centralized aspects, such as salary structures and fundraising, while granting regions autonomy in care coordination, workload management, and scheduling. One physician explained that “*(. . .) day-to-day personnel management should be decentralized. There are certain things in human resources, such as contract and salary negotiations, that should remain centralized.*” *(MDP504)*

Comments from physicians highlight the need for a better understanding of local needs:

We made some recommendations. *One* of the problems is the lack of replacement for staff on leave, which often leads to tasks being postponed or neglected. Things don't move forward, and you have no way of knowing where they’re at if you don't follow up yourself. As a result, our suggestions are put on the back burner, or remain on the shelf, or go unanswered. It's a question of standardization across all hospitals, although you can't compare every hospital. There are challenges that are specific to each hospital, and you can't standardize all of them. There's a certain part of management that must return to being localized to deal with the challenges we face within an organization. (MDP401)

We want familiarity and local power so that people can not only be accessible but also give the impression that they too can make a difference. There’s no need to consult 10 committees to be able to decide. We made the decision, and I'll help you execute it. And the satisfaction, in the “middle management” let's say, they can also be satisfied that they can build something. . . The kind of small communities that have good ideas, we must give them the means. If we give them the means, the confidence and the ability to accomplish these tasks, we'll probably have a much more effective system than the desire for control from above, where everything is the same and we all do the same things. (MDP255)

The participants in our study expressed that decisions affecting them were made without considering their local needs. This contributes to professional dissatisfaction within the RHA and undermines their engagement. When discussing needs, participants refer not only to those of their respective units or hospitals, but also to the communities they serve. They believe that the push to standardize and merge certain processes across the RHA disregards and disrupts local needs. This contributes to a sense of disempowerment among the interviewed professionals and serves as a barrier to their engagement, which may affect their retention within the organization.

### Culture of Openness and Perceived Loss of Control

Many physicians express frustration and a sense of loss of control because of the slow pace of change. The words of one physician exemplify strained relationships:

In family medicine, we are accountable to leaders elsewhere in the network, such as in the region [x]. However, conflicts between regions create tensions. Regions [a, b, c] often harbor a negative perception of regions [x, y] due to their greater resources and access. So, if the rest of us complain about wanting something, it's frustrating for them because they have less. You're only as strong as your weakest link. This desire for uniformity seems to stem from a reluctance to allow some regions to excel beyond others. (MDP416).

A nurse shared their perception of a process lacking communication within the RHA, along with their observations of organizational changes impacting their practice and sense of belonging.

They don't want you to know how things work in *one* region. Just because *one* region can do it, doesn't mean it should be the same everywhere. Often, initiatives are implemented with certain expectations, only to change again, or it's decided that another priority is more important, and that's normal, but transparency is lacking. Communication is inconsistent, leaving staff feeling excluded. I think it's often inhumane what they ask of us. We [nurses] are numbers. It's like all the services are left to themselves, to see that it works. I put the blame higher up [upper management]. (IIP222)

Several participants highlighted the challenge of practicing collaborative leadership within the current context of mistrust beyond the RHA. Despite the RHA’s efforts to improve collaboration, teamwork, and the work environment, challenges persist regarding transparency and trust, which go beyond bureaucratic processes. A recurring theme across our findings was the impact of the 2008 healthcare system reform in New Brunswick, particularly the consolidation of 8 RHAs into 2. This structural change has created a pervasive sense of lost control and uncertainty among healthcare professionals. Such a context directly affects their job satisfaction and commitment, ultimately influencing the RHA’s ability to retain its workforce over time. The following was obtained from a focus group with a managerial team:

So, at the level of the healthcare network - we're talking about nurses here, but at the level of all managers - we're working on the fact that employees are an asset. We need to listen, and we need employees to feel useful, to give meaning to their work, to feel involved, and to feel recognized. People aren't used to that, and I have the impression that in sectors where this change in culture is more widespread, where managers are more open to it, the turnover rate will probably be lower than in other sectors. (FG601)

## Discussion

### Health System Reforms and Administrative Overloads

A key strength of this study lies in its ability to capture the perceptions and experiences of healthcare professionals and evaluate the real impacts of health system reform on their retention within their unique regions and organizational contexts. Participants shared their everyday realities as clinicians, where they are expected to identify medical issues and propose interventions that fit the organizational context of care. Three main themes emerged from our study and highlight the complex interplay between provincial governance, RHA-level decisions and clinicians’ daily work: (a) organizational accountability and frustration, (b) local autonomy and contextual responsiveness, and (c) a culture of openness and perceived loss of control. Usher et al^46^ noted that while the standardization of practices may be a positive aspect of government-initiated reforms, it is countered by reduced accountability^18^ and trust^47^ and increased administrative burdens.^6,14-16^ These burdens hinder the ability of physicians and nurses to work effectively, as transparency can become compromised, and accountability can be challenging. In the case of NB, key decisions related to patient care remain centralized at the steering level. This centralization limits the RHAs’ autonomy in professional matters such as nurse seniority and the allocation of resources for physicians.

### Policy Changes: From “Macro-level” Decisions to Local Realities

The health regions served by the RHA have different needs, contexts, and realities. The centralization efforts that occur at the steering and subsequently at the RHA level tend to dissipate these differences. The development and implementation of future health reforms must consider local characteristics which affect healthcare professional practices and satisfaction, especially in French-speaking minority and rural contexts. Although healthcare systems are structured around a broad policy framework that relies on the will and capacity of political actors,^48^ the local commitment of clinicians and the communities they serve appears essential to the effective implementation of policy change.

The failure to consider local needs leads to frustration and a lack of trust among those affected by the reform, particularly health professionals who feel excluded from decision-making processes and under-equipped to provide adequate care. These findings are consistent with previous research, which demonstrates how top-down approaches particularly affect physicians and nurses in rural areas by creating a disconnect between senior management and professionals in rural communities.^49^

Participants in our study indicated that factors affecting their workload included unclear hierarchical links and the numerous steps required to obtain answers to their questions. These challenges highlight the importance of organizational clarity and responsiveness. To improve retention of physicians and nurses, RHAs should focus on the stability and long-term impact of organizational changes, rather than continuously modifying structures or organizational charts. Continuous restructuring can exacerbate feelings of instability and uncertainty, ultimately undermining workplace culture and professional engagement.^50,51^

### Bureaucracy, Healthcare, and Policymakers

Our main contribution to the literature is the connection between health system reforms and the daily realities of RHAs responsible for delivering health services to meet population needs, while centrally controlling operational structures. These dynamics directly affect the ability of physicians and nurses to provide effective patient care. Our findings suggest that policymakers should recognize that significant changes can have long-lasting effects on the satisfaction and well-being of healthcare professionals. Considering the current shortage of professional healthcare resources, it is strategically prudent for policymakers to focus on strengthening existing foundations of trust, interprofessional collaboration, and workplace stability rather than pursuing further large-scale reforms. There is a perception that RHAs are “big bureaucratic machines,”^52^ which leads to frustration, impersonal work environments, and communication challenges. Policymakers must recognize that exercising collaborative leadership and promoting the satisfaction and well-being of physicians and nurses are extremely challenging in the context of mistrust.

The rigidity of bureaucratic routines, stemming from impersonal rules that define roles in exhaustive detail, often fails to align with the everyday needs of local healthcare professionals. These rules are established at the top of the hierarchy and do not consider the functional goals of the organizations; hence, their impersonal character strays from everyday healthcare functionalities.^49^ Unaddressed areas of uncertainty by these impersonal rules will likely lead to the creation of new rules in an attempt to resolve the issues, but this will only exacerbate the problem, creating a vicious cycle. Therefore, the government must adapt and consider the scale and pace of policy change to prevent unwanted turnover of healthcare professionals such as physicians and nurses.^48^ These changes should be tailored to meet regional needs by allowing for structural and system differences, fostering innovation, and enhancing access to care through collective initiatives and some degree of workplace autonomy. There is a potential for improvement and transformation and suggesting opportunities such as innovative approaches to care and technology-based service delivery models in healthcare.

## Limitations

The current research results are presented as themes, which do not allow us to determine the relative importance of each factor in the context of the well-known difficulty of retaining healthcare professionals in urban and rural areas. The data collected for this study was synchronic and solely based on the opinions of the participants. It was only possible to gather the perspectives of employees (ie, doctors and nurses) and not those of their direct supervisors. This limitation could be an interesting avenue for further research. Establishing partnerships among various health-related stakeholders and understanding the viewpoints of healthcare professionals, their supervisors, and facility management could enrich future research and potentially inform collaborative strategies to address the retention challenges in these regions.

Despite the large and diverse sample size, the focus on exploring nuances may not have captured the breadth needed to generalize our findings. However, they did contribute to existing knowledge about factors that could potentially inhibit the retention of physicians and nurses in healthcare. Another limitation of this study is its cross-sectional (exploratory) design, which did not allow for tracking the evolution of the issue over time but provided a contextual snapshot. It also does not determine which identified factors are the most important or influential.

## Conclusions

Healthcare systems face mounting pressures from increasingly complex care needs, rising chronic and infectious diseases, and growing cultural and linguistic diversity among patients. These challenges strain healthcare delivery and impact the ability of nurses and physicians to provide timely, culturally competent care. Static reforms are no longer sufficient; adaptive, context-sensitive policies grounded in the lived experiences of patients and providers are essential. In this study, physicians and nurses reported confusion over shifting hierarchies and a perceived loss of control, which they felt compromised care quality. These insights highlight the need for reforms that bridge macro-level policy with frontline realities. Effective change requires collaboration among governments, RHAs, regulatory bodies, and healthcare professionals to co-create responsive policies. Ongoing dialog, structural support, and regular feedback from providers are critical to improving job satisfaction, retention, and positive care outcomes. Health system reforms must remain flexible to meet the evolving needs of both professionals and the communities they serve.

## Supplemental Material

sj-docx-1-inq-10.1177_00469580251365821 – Supplemental material for From Policy to Practice: A Qualitative Study on Reforms and Frontline Retention in Healthcare Supplemental material, sj-docx-1-inq-10.1177_00469580251365821 for From Policy to Practice: A Qualitative Study on Reforms and Frontline Retention in Healthcare by Anik Dubé, Stéphanie Collin, Jennifer Hakim, Claire Johnson, Marie-Eve Laforest, Michel H. Landry and Martin Lauzier in INQUIRY: The Journal of Health Care Organization, Provision, and Financing

sj-docx-2-inq-10.1177_00469580251365821 – Supplemental material for From Policy to Practice: A Qualitative Study on Reforms and Frontline Retention in Healthcare Supplemental material, sj-docx-2-inq-10.1177_00469580251365821 for From Policy to Practice: A Qualitative Study on Reforms and Frontline Retention in Healthcare by Anik Dubé, Stéphanie Collin, Jennifer Hakim, Claire Johnson, Marie-Eve Laforest, Michel H. Landry and Martin Lauzier in INQUIRY: The Journal of Health Care Organization, Provision, and Financing

sj-docx-3-inq-10.1177_00469580251365821 – Supplemental material for From Policy to Practice: A Qualitative Study on Reforms and Frontline Retention in Healthcare Supplemental material, sj-docx-3-inq-10.1177_00469580251365821 for From Policy to Practice: A Qualitative Study on Reforms and Frontline Retention in Healthcare by Anik Dubé, Stéphanie Collin, Jennifer Hakim, Claire Johnson, Marie-Eve Laforest, Michel H. Landry and Martin Lauzier in INQUIRY: The Journal of Health Care Organization, Provision, and Financing
