# Supplementary material for: From Policy to Practice: A Qualitative Study on Reforms and Frontline Retention in Healthcare
Source: Inquiry. 2025 Aug 16;62:00469580251365821. doi: 10.1177/00469580251365821 (PMC12357990; doi:10.1177/00469580251365821)
Supplement: sj-docx-2-inq-10.1177_00469580251365821 – Supplemental material for From Policy to Practice: A Qualitative Study on Reforms and Frontline Retention in Healthcare [file sj-docx-2-inq-10.1177_00469580251365821.docx]

**GROUP DISCUSSION GUIDE (CIHR)**

Addressing the Shortage of Health Professionals in CLOSMs

Strengthening Retention Strategies for the Benefit of Francophone and Acadian Communities in New Brunswick

**Introductory Remarks**

- Introductory Remarks
- Thank the participants for joining the discussion.
- Remind them of the study's objectives.
- Emphasize that there are no right or wrong answers. Explain that we are here to listen because we want to better understand their experience with the healthcare system.
- Explain that we aim to give each participant equal speaking time, as much as possible.
- Mention that the discussion will be recorded, but any identifying information will be removed during transcription. Also explain that notes will be taken to help revisit certain points.
- Inform participants that they can leave the discussion group at any time without needing to explain. They are also not required to answer any questions that make them uncomfortable.
- Explain that everything said in the discussion is confidential, meaning no one should talk about the interview afterward.
- Ask if participants have any questions and respond to them.
- Ensure signed consent forms have been collected and confirm permission to record the session.
- Clarify that we want the tone of the discussion to remain informal.

________________________________________

**Discussion**

This section aims to identify participants' perceptions regarding:

Section I – Main challenges encountered

Section II – Known factors impacting retention

Section III – Efforts/measures implemented by the Network to ensure retention

Section IV – Conclusion

**Section I – Challenges (10 minutes)**

Can you tell us about the main challenges you face regarding retention?

Are there specific areas/departments where retention is particularly difficult or others where it's less of a challenge?

________________________________________

**Section II - Retention Factors (20 minutes)**

What makes the Network a good or bad employer?

Can you tell us about the measures used to learn more about staff retention? Do you have ways to understand employees' perceptions of the work environment, job satisfaction, etc.?

In your opinion, what are the key retention factors for professionals in the Network? (For example, how much value is placed on career development?)

________________________________________

**Section III - Efforts and Measures (15 minutes)**

Can you tell us about activities or programs implemented to support recruitment or retention within the Network?

Can you tell us about activities or programs aimed at ensuring employee satisfaction?

How do you involve CLOSMs in recruitment/retention efforts? (For us: Do you think their specific health needs are being met? How does the Network view its role/commitment in improving access to care for CLOSMs?)

How do you perceive your relationships with the Department of Health and other stakeholders (such as educational institutions, NGOs) when it comes to recruitment and retention? (Are financial resources sufficient to ensure adequate staffing?)

**Section IV – Conclusion (10 minutes)**

Would anyone like to add anything before we end the discussion?

What stood out to you? What was most important to you during this discussion?

**Thank you very much for your participation and honesty.**
