# Supplementary material for: From Policy to Practice: A Qualitative Study on Reforms and Frontline Retention in Healthcare
Source: Inquiry. 2025 Aug 16;62:00469580251365821. doi: 10.1177/00469580251365821 (PMC12357990; doi:10.1177/00469580251365821)
Supplement: sj-docx-3-inq-10.1177_00469580251365821 – Supplemental material for From Policy to Practice: A Qualitative Study on Reforms and Frontline Retention in Healthcare [file sj-docx-3-inq-10.1177_00469580251365821.docx]

**INTERVIEW GUIDE (CIHR)**

Addressing the Shortage of Health Professionals in CLOSMs

Strengthening Retention Strategies for the Benefit of Francophone and Acadian Communities in New Brunswick

**Identification**

| **Date:**  **Form Number:** |
| --- |

**Foreword**

- Remind participants of the research project and its objectives.
- Present the ethical guidelines and other procedures:
- Informed consent
- Protection of anonymity
- Proposal to record the interview
- Option to stop the recording at any time upon request
- Present the consent form (previously sent by email).
- Obtain the participant’s signature on the consent form.

**Section 1: Introduction**

1. Can you briefly describe your professional background?

**Section 2: Structure/Organization**

1. To what extent do you feel you have control or influence over decisions that involve you, and that your needs are heard and respected?
2. What factors influence your satisfaction in your current position?
3. How do you perceive your workload and the demands associated with your job?

**Section 3: Transformational Leadership**

1. How would you describe your supervisor’s leadership style?
2. What is the quality of the relationships among members of your team?

**Section 4: Exemplary Professional Practices and Innovations**

1. How much value is placed on professional development within your team?
2. Do you feel you have autonomy or some freedom to improve the quality of care you provide to your patients?
3. To what extent was the working language a determining factor in your choice of employer?

9.b How is language associated with your decision to stay in your position or to leave?

**Conclusion**

1. What might lead you to stay in your position or to leave your job?
2. Finally, is there anything else you would like to add or clarify regarding the points we’ve just discussed?

**Thank you very much for your participation and honesty.**
